# Serum miR-373-3p and miR-194-5p Are Associated with Early Tumor Progression during FOLFIRINOX Treatment in Pancreatic Cancer Patients: A Prospective Multicenter Study

**DOI:** 10.3390/ijms222010902

**Published:** 2021-10-09

**Authors:** Fleur van der Sijde, Marjolein Y. V. Homs, Marlies L. van Bekkum, Thierry P. P. van den Bosch, Koop Bosscha, Marc G. Besselink, Bert A. Bonsing, Jan Willem B. de Groot, Thomas M. Karsten, Bas Groot Koerkamp, Brigitte C. M. Haberkorn, Saskia A. C. Luelmo, Leonie J. M. Mekenkamp, Dana A. M. Mustafa, Johanna W. Wilmink, Casper H. J. van Eijck, Eveline E. Vietsch, on behalf of the Dutch Pancreatic Cancer Group

**Affiliations:** 1 Department of Surgery, Erasmus MC Cancer Institute, University Medical Center Rotterdam, 3000 CA Rotterdam, The Netherlands; f.vandersijde@erasmusmc.nl (F.v.d.S.); b.grootkoerkamp@erasmusmc.nl (B.G.K.); e.vietsch@erasmusmc.nl (E.E.V.); 2 Department of Medical Oncology, Erasmus MC, University Medical Center Rotterdam, 3000 CA Rotterdam, The Netherlands; m.homs@erasmusmc.nl; 3 Department of Medical Oncology, Reinier de Graaf Gasthuis, 2625 AD Delft, The Netherlands; M.vanBekkum@rdgg.nl; 4 Department of Pathology, Erasmus MC, University Medical Center Rotterdam, 3000 CA Rotterdam, The Netherlands; t.vandenbosch@erasmusmc.nl; 5 Department of Surgery, Jeroen Bosch Hospital, 5223 GZ ‘s Hertogenbosch, The Netherlands; K.Bosscha@jbz.nl; 6 Department of Surgery, Cancer Center Amsterdam, Amsterdam UMC, University of Amsterdam, 1105 AZ Amsterdam, The Netherlands; m.g.besselink@amsterdamumc.nl; 7 Department of Surgery, Leiden University Medical Center, 2333 ZA Leiden, The Netherlands; B.A.Bonsing@lumc.nl; 8 Isala Oncology Center, Isala Hospital, 8025 AB Zwolle, The Netherlands; j.w.b.de.groot@isala.nl; 9 Department of Surgery, Onze Lieve Vrouwe Gasthuis, 1061 AE Amsterdam, The Netherlands; t.m.karsten@olvg.nl; 10 Department of Medical Oncology, Maasstad Hospital, 3079 DZ Rotterdam, The Netherlands; HaberkornB@maasstadziekenhuis.nl; 11 Department of Medical Oncology, Leiden University Medical Center, 2333 ZA Leiden, The Netherlands; S.A.C.Luelmo@lumc.nl; 12 Department of Medical Oncology, Medisch Spectrum Twente, 7512 KZ Enschede, The Netherlands; L.Mekenkamp@mst.nl; 13 Tumor Immuno-Pathology Laboratory, Department of Pathology, Erasmus MC, University Medical Center Rotterdam, 3000 CA Rotterdam, The Netherlands; d.mustafa@erasmusmc.nl; 14 Department of Medical Oncology, Cancer Center Amsterdam, Amsterdam UMC, University of Amsterdam, 1105 AZ Amsterdam, The Netherlands; j.w.wilmink@amsterdamumc.nl

**Keywords:** pancreatic cancer, FOLFIRINOX, predictive biomarker, miR-373, miR-194

## Abstract

In this study, we explored the predictive value of serum microRNA (miRNA) expression for early tumor progression during FOLFIRINOX chemotherapy and its association with overall survival (OS) in patients with pancreatic ductal adenocarcinoma (PDAC). A total of 132 PDAC patients of all disease stages were included in this study, of whom 25% showed progressive disease during FOLFIRINOX according to the RECIST criteria. MiRNA expression was analyzed in serum collected before the start and after one cycle of chemotherapy. In the discovery cohort (*n* = 12), a 352-miRNA RT-qPCR panel was used. In the validation cohorts (total *n* = 120), miRNA expression was detected using individual RT-qPCR miRNA primers. Before the start of FOLFIRINOX, serum miR-373-3p expression was higher in patients with progressive disease compared to patients with disease control after FOLFIRINOX (Log2 fold difference (FD) 0.88, *p* = 0.006). MiR-194-5p expression after one cycle of FOLFIRINOX was lower in patients with progressive disease (Log2 FD −0.29, *p* = 0.044). Both miRNAs were predictors of early tumor progression in a multivariable model including disease stage and baseline CA19-9 level (miR-373-3p odds ratio (OR) 3.99, 95% CI 1.10–14.49; miR-194-5p OR 0.91, 95% CI 0.83–0.99). MiR-373-3p and miR-194-5p did not show an association with OS after adjustment for disease stage, baseline CA19-9, and chemotherapy response. In conclusion, high serum miR-373-3p before the start and low serum miR-194-5p after one cycle are associated with early tumor progression during FOLFIRINOX.

## 1. Introduction

Pancreatic ductal adenocarcinoma (PDAC) is an aggressive cancer associated with poor prognosis [1]. Its incidence is rising and predictions show that PDAC could become the second leading cause of cancer-related deaths by 2030 [2]. Unfortunately, there has been only a slight improvement in the treatment of PDAC in the last decennium. Most patients will receive chemotherapy, of which FOLFIRINOX, a combination of fluorouracil, leucovorin, irinotecan and oxaliplatin, is one of the most effective and most commonly used regimens. First-line FOLFIRINOX prolongs survival of advanced PDAC patients [3,4] as well as survival in patients with resectable or borderline resectable disease when administered as adjuvant treatment [5]. Although FOLFIRINOX will stabilize the disease in most patients, median overall survival (OS) is still only 11 months in patients with metastatic PDAC [4]. Furthermore, patients often experience severe chemotherapy-induced toxicity [3,4,5,6].

Chemotherapy resistance is one of the main reasons for the lack of survival benefit. In PDAC, both intrinsic and acquired mechanisms play a role in chemotherapy resistance [7]. Intrinsic chemotherapy resistance is caused by the dysregulation of the tumor microenvironment: PDAC tumors are surrounded by a dense stromal layer and are poorly vascularized, both leading to hypoxia in the tumor, and PDAC tumors are rich in tumor-promoting, immunosuppressive components [7,8,9]. Acquired chemotherapy resistance is the result of selection of cancer cells with mechanisms leading to insensitivity to chemotherapy. These include, for example, insensitivity to chemotherapy-induced apoptosis, increased DNA repair mechanisms, dysregulation of the cell cycle, and multidrug resistance caused by increased expression of cell membrane transporters that eliminate chemotherapeutic drugs from the cell [7,9,10].

Many of these chemotherapy resistance processes are regulated by non-coding RNAs [10,11]. These microRNAs (miRNAs) are important post-transcriptional regulators of gene expression by modulation of target messenger RNA (mRNA). A single miRNA can regulate multiple genes, and one gene can be influenced by many different miRNAs. Therefore, miRNAs can alter most cellular processes, including processes initiating chemotherapy resistance [10,11].

Dysregulation of miRNA expression could potentially predict chemotherapy resistance and guide patient selection for chemotherapy-based treatment, such as FOLFIRINOX [10,12]. In this study, we measured circulating miRNA expression in serum before and after one cycle of treatment in PDAC patients with disease control and progressive disease after FOLFIRINOX. MiRNA expression was measured using reverse transcription quantitative polymerase chain reaction (RT-qPCR). Additionally, we explored the predictive value of serum miRNAs for early disease progression and their prognostic value for overall survival (OS).

## 2. Results

### 2.1. Patient Characteristics

For the three cohorts combined, the discovery cohort (*n* = 12), validation cohort 1 (*n* = 60), and validation cohort 2 (*n* = 60), a total of 132 patients were selected for circulating miRNA analysis. Patient characteristics are presented in Table 1. Almost half of the patients (47.0%) presented with resectable or borderline resectable PDAC, 34.1% with locally advanced pancreatic cancer (LAPC), and 18.9% with metastatic PDAC. The percentage of patients with resectable or borderline resectable disease was higher in the validation cohorts compared to the discovery cohort (*p* = 0.016), due to the low availability of samples at the beginning of the PREOPANC-2 trial. There were no other significant differences in patient characteristics between cohorts.

### 2.2. Serum miRNA Expression in the Discovery Cohort

**Figure 1:**
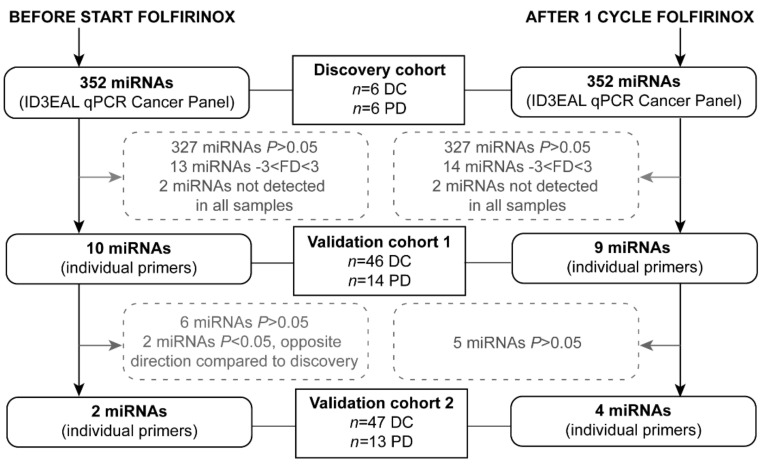
Flowchart of the selection of microRNAs from the discovery panel for the two validation cohorts. DC = disease control, FD = fold difference, PD = progressive disease.

A step-wise approach, shown in Figure 1, was used to select serum miRNAs of interest from an exploratory screening panel. In the discovery cohort, consisting of six disease control patients and six patients with progressive disease after FOLFIRINOX treatment, 352 miRNAs were analyzed. The miRNAs miR-26a-5p and miR-30b-5p combined showed the best stability value based on 24 discovery samples (twelve before the start of FOLFIRINOX and twelve after one cycle of FOLFIRINOX) and were selected as reference miRNAs for all cohorts.

**Figure 2:**
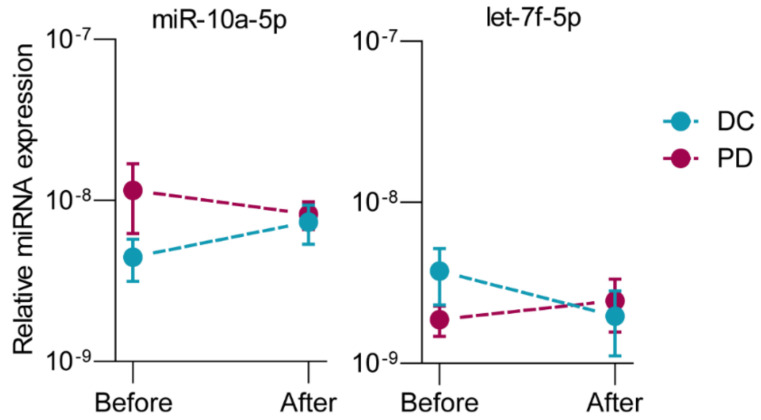
Changes in serum miRNA expression over time measured before start of FOLFIRINOX and after one cycle of FOLFIRINOX with opposite directions between patients with disease control (DC, *n* = 6) and progressive disease (PD, *n* = 6) in the discovery cohort. Data are presented as average FD, relative to the expression of the two reference miRNAs, with standard deviations. *p*-values were calculated with paired *t*-tests.

Before the start of FOLFIRINOX, ten miRNAs from the 352-miRNA panel showed a statistically significant fold difference between patients with disease control and patients with progressive disease, as shown in Table 2. From these ten, four miRNAs were upregulated, and six were downregulated in patients with progressive disease compared to patients with disease control. After one cycle of FOLFIRINOX, nine miRNAs showed a significant fold difference (Table 2). All nine miRNAs were downregulated in patients with progressive disease compared to patients with disease control after FOLFIRINOX. The miRNAs let-7g-5p, miR-194-5p, miR-30a-5p were both before and after one cycle of FOLFIRINOX downregulated in patients with progressive disease. MiR-10a-5p and let-7f-5p showed opposite changes in expression after one cycle of FOLFIRINOX between patients with disease control and patients with progressive disease. In patients with disease control, miR-10a-5p showed an increase (Log2 fold of change (FOC) 0.78) in expression after one cycle of chemotherapy, while a decrease (Log2 FOC −0.22) in progressive disease patients (*p* = 0.006, Figure 2A). Let-7f-5p showed a decrease (Log2 FOC −0.80) after one cycle of chemotherapy in disease control and increase (Log2 FOC 0.52) in progressive disease patients (*p* = 0.046, Figure 2B).

A total of eighteen differently expressed miRNAs from the discovery panel, before the start of FOLFIRINOX, after one cycle of FOLFIRINOX, or both, and those with different expression patterns over time were selected for validation cohort 1. 

### 2.3. Serum miRNA Expression in the Validation Cohorts

The results of both validation cohorts are presented in Table 2. 

In validation cohort 1, only miR-17-3p remained statistically significantly expressed between disease control and progressive patients before the start of FOLFIRINOX (Log2 FD 0.49, *p* = 0.048). Two miRNAs, miR-30a-5p and miR-629-5p, also showed a significantly higher expression in progressive disease patients in validation cohort 1. Contrarily, these same miRNAs were downregulated in the discovery cohort and therefore not selected for further evaluation. After one cycle of FOLFIRINOX, miR-18a-5p (Log2 FD −0.32, *p* = 0.027), miR-194-5p (Log2 FD −0.50, *p* = 0.026), miR-24-3p (Log2 FD −0.78, *p* = 0.024), and miR-27a-3p (Log2 FD −0.95, *p* = 0.008) remained significantly downregulated in patients with progressive disease compared to patients with disease control. These five statistically significant miRNAs (one before start of FOLFIRINOX, four after one cycle of FOLFIRINOX) were selected for additional validation in validation cohort 2. In addition, miR-373-3p was also selected for validation cohort 2. MiR-373-3p was the miRNA with the highest fold difference in the discovery cohort and therefore a promising predictive biomarker. Additionally, it has been reported as an important cancer miRNA in the literature. The raw threshold cycle (Ct) values of miR-373-3p were significantly lower in validation cohort 1, suggesting a technical difference between the 352-miRNA discovery panel and the individual primers.

In validation cohort 2, miR-373-3p (Log2 FD 0.92. *p* = 0.007) before the start of FOLFIRINOX, and miR-18a-5p (Log2 FD 0.56, *p* = 0.016) after one cycle of FOLFIRINOX showed significant FD between progressive disease compared to patients with disease control (Table 2). However, while miR-18a-5p was upregulated in validation cohort 2, it was downregulated in validation cohort 1. Due to this discrepancy, we did not further investigate miR-18a-5p.

When combining the results of both validation cohorts, miR-373-3p (Log2 FD 0.88, *p* = 0.006) before the start of FOLFIRINOX, and miR-194-5p (Log2 FD −0.29, *p* = 0.044) after one cycle of FOLFIRINOX remained significantly differently expressed between patients with progressive disease and patients with disease control (Table 2). 

In a multivariable model, miR-373-3p expression before therapy (OR 3.99, 95% CI 1.10–14.49, *p* = 0.035) and miR-194-5p expression after one cycle of FOLFIRINOX (OR 0.91, 95% CI 0.83–0.99, *p* = 0.030) remained significant predictive factors of early tumor progression during FOLFIRINOX (Table 3). Expression of miR-373-3p and expression of miR-194-5p were not correlated (Pearson’s *r* = 0.032, *p* = 0.742).

### 2.4. Serum miRNA Expression between Disease Stages

No difference in serum miRNA expression was found between stages of disease for any of the miRNAs from the validation cohorts, except for miR-17-3p (Table S1). MiR-17-3p was overexpressed in resectable disease patients with early tumor progression during FOLFIRINOX compared to resectable patients with disease control (Log2 FD 0.58, *p* = 0.040, Table S2). This difference in miR-17-3p expression was not found in patients with LAPC or metastatic disease. 

### 2.5. Serum miRNA Expression and Overall Survival

The median follow-up time was 14.0 months for patients alive at last follow-up. The median OS for the total cohort of PDAC patients was 11.7 months. In univariable analyses, miR-373-3p and miR-194-5p expression were not associated with OS. Serum miR-17-3p expression before start of FOLFIRINOX was a prognostic factor for OS (HR 1.30, 95% CI 1.02-1.65, *p* = 0.032), as shown in Table 4. However, in multivariable analysis, after adjustment for stage of disease, baseline CA19-9 level, and RECIST chemotherapy response outcome, miR-17-3p expression did not remain a significant predictor of OS (HR 1.18, 95% CI 0.92-1.52, *p* = 0.192).

### 2.6. Tissue miR-373-3p Expression

**Figure 3:**
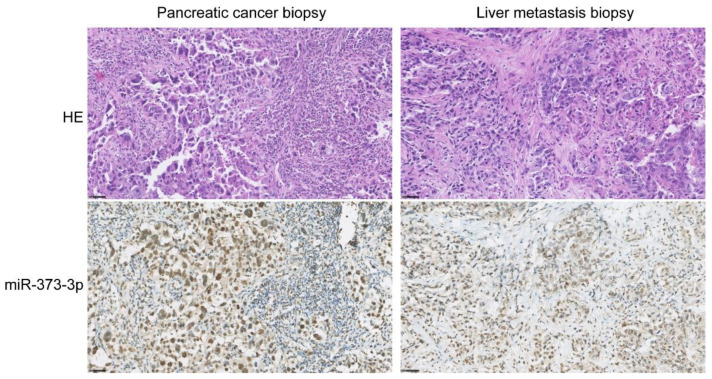
In situ hybridization of treatment-naïve pancreatic ductal adenocarcinoma (PDAC) and PDAC metastasis biopsies for miR-373-3p. Tissue sections from a primary PDAC tumor and from a PDAC liver metastasis (before FOLFIRINOX) are stained with hematoxylin and eosin (HE) and with miR-373-3p, visualized with DAB. Scale bar = 100 µm.

To assess the origin of serum miR-373-3p expression, treatment-naïve PDAC and PDAC metastasis tissue biopsies were analyzed by in situ hybridization (ISH) with miR-373-3p probes. Positive and negative control tissue staining with U6 and scramble miRNA probes is visualized in Figure S1. The miR-373-3p expression in healthy tissues is shown in Figure S2. MiR-373-3p is expressed by normal endothelium, colon epithelial cells, hepatocytes, renal tubular cells, neurons, and tonsillar B cell lymphoid follicles. Lung epithelium and T cells do not express detectable levels of miR-373-3p. In normal pancreatic tissue, miR-373-3p is expressed in acinar cells only, pancreatic ductal cells do not express miR-373-3p (Figure S1). However, pancreatic ductal adenocarcinoma cells, both in primary PDAC tissues as well as PDAC liver metastases, express high levels of miR-373-3p (Figure 3). 

As tumor samples after one cycle of treatment were not available, miR-194-5p was not further investigated by ISH, since this miRNA showed predictive value during FOLFIRINOX treatment instead of before the start of treatment.

## 3. Discussion

In this multicenter, prospective study we found that PDAC patients with progressive disease compared to patients with disease control showed higher expression of serum miR-373-3p before the start of FOLFIRINOX and lower expression of serum miR-194-5p after one cycle of FOLFIRINOX. In multivariable logistic regression, both miRNAs were significant predictors of early tumor progression during FOLFIRINOX. Expression of serum miR-373-3p and miR-194-5p was not associated with OS.

Studies investigating circulating miRNAs in PDAC patients undergoing chemotherapy are scarce and currently no miRNA biomarker has been validated for clinical use. Several studies have been published on the diagnostic [13,14,15] and prognostic value [15,16,17,18] of circulating miRNAs. The present study is one of the first on circulating miRNAs and their value to predict treatment outcome in patients with PDAC. A previous study by Meijer et al. showed that patients with early progression after completion of FOLFIRINOX treatment overexpress plasma miR-181a-5p after treatment compared to patients with longer progression-free survival, as measured with RT-qPCR [12]. In our discovery cohort, miR-181a-5p did not show a difference in expression between patients with disease control and patients with progressive disease during FOLFIRINOX. However, an important difference between the two studies is the time point of blood collection and miRNA measurement. Meijer et al. collected plasma samples from 54 advanced PDAC patients after 5–6 cycles of FOLFIRINOX [12], while we collected blood for serum miRNA expression analysis after only one cycle of FOLFIRINOX. The more cycles of chemotherapy patients receive, the more evident the differences in biological (non)response to treatment will become. In contrast, we measured miRNA expression before the start and in an early phase of treatment; a predictive biomarker before chemotherapy or after one cycle can better guide subsequent treatment. 

Other miRNAs have been described for their role in chemotherapy response [19,20]. For example, miR-200b, miR-200c, and miR-21 were often found to be involved in 5-FU resistance [19]. Unfortunately, these findings on miRNAs influencing chemotherapy response are based on research performed in cell lines [19], which do not resemble pancreatic tumor tissue from patients. Cell lines might undergo genotypic and phenotypic transformation and are not exposed to cancer-associated environmental components, including stromal and immunological factors [21]. Especially in miRNA research, the interaction with tumor stroma is important, since a large part of circulating miRNAs originate from endothelium and immune cells and circulating miRNAs often target messenger RNAs involved in immune responses [22,23].

In this study, we found serum miR-373-3p and miR-194-5p to be associated with early tumor progression during FOLFIRINOX. Two studies both showed downregulation of miR-373-3p in PDAC patients compared to healthy individuals in tissue [24] and serum samples [25]. Lower expression was also associated with poor prognostic clinical features and OS [25]. These findings are inconsistent with the results of the present study in which upregulation of miR-373-3p was associated with early tumor progression. The difference in miR-373 expression could lie in differences between the patient populations. The description of patient characteristics in these two studies, however, is insufficient to allow for comparison. Our ISH results show that miR-373-3p is expressed by normal acinar cells, and highly expressed by PDAC cells, whereas no expression was detected in the tumor stroma, which is the largest component of PDAC tumors. RNA from homogenized tumor tissue does not allow for cell-specific miRNA measurements, which might explain differences between our findings and those described in the literature. On the other hand, miR-373-3p is known to act in an ambiguous way; in some cancers this miRNA has been described as a tumor suppressor (e.g., in perihilar cholangiocarcinoma), while in other cancers miR-373 shows tumor promoting properties (e.g., in hepatocellular carcinoma and breast cancer) [26]. Many genes have been identified as targets of miR-373-3p [26]. According to the miRNA Target Database (miRDB) [27], miR-373-3p has 899 predicted gene targets, of which the top two are: *YOD1* and *LATS2*. *YOD1* and *LATS2* are cell cycle genes, but *LATS2* is also a regulator of the p53-pathway [28]. One of the most interesting functions of miR-373 in the light of PDAC, is that this miRNA cooperates with oncogenic RAS to overcome the need for P53 loss to achieve cancer cell proliferation, which was demonstrated in testicular germ-cell tumors. The wild-type (WT) *TP53* gene, and its P53 protein, are tumor suppressors inducing cellular senescence. Normally, P53 will have an anti-proliferative response to oncogenic RAS-induced tumorigenesis. However, expression of miR-373 inactivates P53 and therefore the senescence mechanism is bypassed [26,29]. We found high levels of miR-373-3p in PDAC cells, in contrast to normal pancreatic ductal cells that do not show miR-373-3p tissue expression with ISH. This, together with the fact that serum miR-373-3p levels are already higher before the start of treatment in patients with disease progression upon FOLFIRINOX, also suggests that miR-373-3p is a PDAC cell-intrinsic malignant factor and not, for example, an immunologic miRNA in response to PDAC.

*KRAS* and *TP53* are the most frequently mutated genes in PDAC [30]. Investigating if miR-373 levels differ between PDAC patients with WT *TP53* and patients with *TP53* mutations would be an interesting next step. A relative upregulation of miR-373 might only be detected in patients with mutated *KRAS* in combination with WT *TP53*. Unfortunately, the mutational status is unknown for most patients included in our patient cohort.

To our knowledge, miR-194-5p has been reported in PDAC patients only once. This miRNA was overexpressed in PDAC tissue and serum samples compared to healthy controls and ectopic expression of miR-194-5p in PDAC cell lines promoted cell proliferation and migration [31]. This is in contrast to our findings. However, in multiple different cancer experiments, including gastric cancer, lung cancer, and nasopharyngeal cancer, it is shown that miR-194 suppresses cancer cell proliferation, which is in line with our results [32,33,34]. Additionally, in osteosarcoma and colorectal cancer, low serum miR-194 was associated with poor prognosis, comparable to our findings [35,36].

The bidirectional, somewhat ambiguous results in the literature are an important limitation of miRNA research in general. MiRNAs target many mRNAs and proving miRNA involvement in different cellular pathways is challenging. The function of miRNAs differs between tissues and cell types. Moreover, circulating miRNA expression shows a large variation between individuals. In this study, we did not investigate the underlying mechanism of how upregulation of miR-373-3p and downregulation of miR-194-5p in serum may cause early tumor progression during FOLFIRINOX.

A limitation of our study is that we did not include healthy controls and thus we are not able to determine whether serum miR-373-3p and miR-194-5p are differentially expressed compared to healthy individuals. Furthermore, we could not make a differentiation between the different stages of disease of the included patients due to the low numbers in the individual cohorts and response groups. Additionally, because of the relatively low number of patients in the discovery cohort (*n* = 12), other miRNAs of importance might not have reached statistical significance and were therefore not selected for validation.

Further validation of the miRNAs described in this study in a larger patient cohort allows the distinction of subgroups of patients with PDAC, not only based on the stage of disease, but also based on tumor biology and treatment response. We have shown that miR-373-3p and miR-194-5p are significantly different between responding and non-responding patients, already before the start of treatment and after one cycle of FOLFIRINOX. This suggests that response can be determined by using circulating biomarkers much earlier than with CT evaluation. Monitoring circulating miRNA expression could be a tool to select patients for available treatments, to spare patients from ineffective therapy, and to identify potential targets for future therapies.

## 4. Materials and Methods

This article was written according to the Reporting recommendations for tumor marker prognostic studies (REMARK) guidelines [37].

### 4.1. Patient Selection

All patients, initially treated with FOLFIRINOX, were selected from two multicenter, prospective trials in The Netherlands. Patients with resectable or borderline resectable PDAC participated in the randomized clinical trial PREOPANC-2 (Dutch trial register NL7094) comparing neoadjuvant FOLFIRINOX to neoadjuvant gemcitabine-based chemoradiotherapy, followed by surgical resection of the primary tumor if applicable. Patients with locally advanced and metastatic PDAC were selected from the prospective cohort study iKnowIT (Dutch trial register NL7522) investigating the predictive value of circulating biomarkers. The trials were approved by the ethics committees of all participating hospitals: Erasmus MC (ethics committee reference number MEC-2018-087 and MEC-2018-004), Amsterdam UMC (2018_196 and 2018_138), Leiden University Medical Center (L18.070 and L18.053), Isala hospital, Zwolle (180606), Reinier de Graaf Gasthuis, Delft (SK/CS 19-119), Jeroen Bosch hospital, Den Bosch (2018.07.17.01), Maasstad hospital, Rotterdam (L2018053 and L2018095), Onze Lieve Vrouwe Gasthuis, Amsterdam (WO 18.118), and Medisch Spectrum Twente, Enschede (H18-081).

Due to the explorative character of this study, no formal sample size calculation was performed. Patients were selected based on the availability of serum samples and treatment response outcome. 

After histopathological confirmation of the primary tumor and/or metastases, patients from all PDAC disease stages received initial treatment with FOLFIRINOX between February 2018 and November 2020. Patients received a maximum of 12 cycles. Exclusion criteria for patient selection were age under 18 years, WHO performance status >1, and previous treatment with FOLFIRINOX. A staging CT scan was performed a maximum of four weeks prior to the start of chemotherapy. A CT scan to evaluate the tumor response to treatment was performed after every fourth cycle of FOLFIRINOX, or earlier if patients showed clinical signs of tumor progression, according to the Response Evaluation Criteria in Solid Tumours (RECIST) 1.1 criteria [38] as part of standard clinical practice. Final treatment response was defined as the treatment response measured on the CT scan immediately after the last cycle of FOLFIRINOX. In patients with progressive disease, FOLFIRINOX was discontinued. Disease control was defined as stable disease, partial, or complete response. Patients with disease control continued with FOLFIRINOX for a maximum of 12 cycles. Patient characteristics, such as age, sex, stage of disease, laboratory results, CT scan evaluations, and follow-up data were retrieved from medical records by a medical doctor. Follow-up ended upon the death of the patient.

### 4.2. Sample Collection

Peripheral venous blood samples were collected before the start of FOLFIRINOX and two weeks after the first cycle, before start of the second cycle of FOLFIRINOX. Blood was collected in 10 mL serum tubes with clot activator of silica particles (Becton Dickinson, Franklin Lakes, NJ, USA). Within two hours after collection, blood samples were centrifuged for ten minutes at 2000× *g*, and serum was stored at −80 °C until further use.

### 4.3. Serum miRNA Isolation and Quantitation

MiRNAs were analyzed in three cohorts: a discovery cohort, validation cohort 1, and validation cohort 2. Only differentially expressed serum miRNAs between patients with disease control and patients with progressive disease with a fold difference (FD) of ≤0.33 or ≥3 (corresponding to a Log2 FD of ≤−1.59 or ≥1.59) and *p* < 0.05, that were detectable within the raw Ct value limits in all twelve patients from the discovery cohort, were selected for validation cohort 1. MiRNAs that remained statistically significantly up- or downregulated were selected for validation cohort 2. MiRNA selection in the different cohorts is shown in Figure 1. 

RNA was isolated from 2 × 200 µL or 200 µL serum using the miRNeasy serum/plasma miRNA Isolation Kit (Qiagen, Hilden, Germany) for the discovery cohort and validation cohorts, respectively. In the validation cohorts, three proprietary pre-mixed spike-in ~20 nucleotide control RNAs (MiRXES, Singapore) with sequences distinct from annotated mature human miRNAs (miRbase version 21) were added to the lysis buffer prior to the serum miRNA isolation according to the manufacturer’s instructions, in order to evaluate RNA isolation efficiency. 

In the discovery cohort, serum miRNAs were reverse transcribed (RT) using ID3EAL miRNA-specific oligo’s and RT spike-in RNA (MiRXES, Singapore) in a multiplex reaction per manufacturer’s instruction. Complementary DNA (cDNA) was stored at −20 °C up to two weeks and thawed only once. cDNA was added to the ID3EAL miRNA qPCR Master Mix, containing buffer, polymerase and the passive reference dye ROX, and transferred to pre-loaded ID3EAL 384 Target Assay Panel plates (MiRXES, Singapore), including 352 individual quantitative polymerase chain reaction (qPCR) primers, 16 reverse transcription spike-ins and 16 inter-plate controls. PCR amplification was performed with the 7500 Fast Real-Time PCR system (Applied Biosystems, Foster City, CA, USA). Raw Ct values were determined using the 7500 Software (version 2.3; Applied Biosystems, Foster City, CA, USA).

In the two validation cohorts, selected serum miRNAs were measured with ID3EAL RT-qPCR after reverse transcription, using individual miRNA primers (MiRXES, Singapore). Raw Ct values were determined using the 7500 Software (version 2.3; Applied Biosystems, Foster City, CA, USA).

In the discovery cohort, raw Ct values were normalized using reference miRNAs, spike-ins, and inter-plate calibrators, using an algorithm incorporated in the Cancer Panel Analysis Template (version 1.9, MiRXES, Singapore), provided by the manufacturer. The two miRNAs with the most stable expression among all samples (miR-26a-5p and miR-30b-5p) were selected as reference miRNAs, for both the discovery and validation cohorts, using NormFinder software for Excel (version 0.953; MOMA, Aarhus University Hospital, Aarhus, Denmark). The cutoff values for detection of raw Ct values were 9-33 cycles, based on the manufacturer’s recommendations. In the validation cohorts, raw Ct values were normalized using the same two reference miRNAs (miR-26a-5p and miR-30b-5p). Data from the validation cohorts were normalized and analyzed using the online Thermo Fisher Connect Platform (Thermo Fisher Scientific, Waltham, MA, USA).

### 4.4. In Situ Hybridization (ISH) of Pancreatic Cancer Tissue

Diagnostic biopsies of PDAC and PDAC liver metastases were collected at the Erasmus MC for clinical pathology evaluation. Biopsies of primary PDAC tumors were obtained by endoscopic ultrasound-guided fine-needle biopsies. To histopathologically confirm metastatic disease in the liver, fine-needle biopsies were obtained by guidance of ultrasound or CT. Stored formalin-fixed paraffin-embedded (FFPE) tissue blocks were analyzed for clinical histopathological diagnosis and residual material was used for biomarker analysis. Four-micrometer-thick tissue sections were processed in the Discovery Ultra instrument (Ventana Medical Systems, Oro Valley, AZ, USA) with the automated Discovery Universal protocol. In brief, after deparaffinization and heat-induced antigen retrieval with CC1 (#950-124, Ventana) for 16 minutes at 100 °C followed by ISH protease 2 (#780-4148, Ventana) for 4 minutes at 37 °C, peroxidase inhibitor CM (#760-159, Ventana) was added for 8 minutes followed by the addition of 20 nM of 3′ and 5′-DIG labeled miRCURY LNA miRNA miR-373-3p detection probes (Qiagen, Hilden, Germany) for 8 minutes at 84 °C. Hybridization at 55 °C for 1 hour was followed by wash steps with 1xSSC (#950-110, Ventana). Detection with anti-DIG HRP (#760-4822, Ventana) and Disc Amp BF (#760-226, Ventana) was followed by visualization with DAB (#760-159, Ventana). The tissues were counterstained with Hematoxylin II (Ventana). Adjacent tissue sections were stained with hematoxylin and eosin (HE). The slides were scanned using the Nanozoomer 2.0-HT slide imager (Hamamatsu Photonics, Hamamatsu City, Japan).

### 4.5. Statistical Analysis

Patient characteristics were compared between the different cohorts with Kruskall–Wallis tests for continuous data, including age, number of FOLFIRINOX cycles, and baseline CA19-9 levels, and with Chi-squared tests for categorical data: sex, stage of disease, and RECIST response outcome. 

MiRNA expression was analyzed in three ways: differences in miRNA expression before the start of FOLFIRINOX, miRNA expression after one cycle of FOLFIRINOX, and differences in miRNA expression change over time between patients with disease control and patients with progressive disease.

MiRNA expression relative to the reference miRNAs was calculated with the delta Ct method; expression = 2^−(Ct miRNA of interest – average Ct reference miRNAs)^. The mean miRNA expression was compared between patients with disease control and patients with progressive disease using a two-tailed t-test. The disease control patient group was selected as the reference group to calculate fold differences between disease control and progressive disease patients. 

In addition, fold of change (FOC) in miRNA expression within groups after one cycle of FOLFIRINOX was tested with paired t-tests. Statistical significance of FOC over time between disease control and progressive disease patients was compared with two-tailed t-tests. Only miRNAs that showed opposite directions of the FOC in disease control and progressive disease patients, meaning upregulated in one group, downregulated in the other, analyzed with paired t-tests, were found to be clinically significant and therefore selected for further analyses. Differences in miRNA expression between stages of disease were tested with one-way ANOVA. Correlations between expression of different miRNAs were tested with Pearson’s correlation. 

Univariable and multivariable binary logistic regression was performed to analyze the predictive value of relative miRNA expression and known predictive tumor characteristics: stage of disease, and baseline serum CA19-9 level. Variables with *p* < 0.10 were selected for multivariable analysis.

Overall survival (OS) was calculated as the time between the start of FOLFIRINOX and death. The prognostic value of circulating miRNA expression was tested with univariable and multivariable Cox regression analysis, including known prognostic factors: age, stage of disease, chemotherapy response, and baseline serum CA19-9 level.

Statistical analyses were performed with the online Thermo Fisher Connect Platform (Thermo Fisher Scientific, Waltham, MA, USA) and SPSS Statistics for Windows (version 25.0; IBM, Armonk, NY, USA). *p*-values <0.05 were considered statistically significant.

## 5. Conclusions

High expression of miR-373-3p before the start of FOLFIRINOX and low expression of miR-194-5p after one cycle of FOLFIRINOX are associated with early tumor progression during treatment, but do not correlate with OS. This research shows new insights in the progression of PDAC, future clinical utility of miRNAs as predictive biomarkers, and possibly new therapeutic targets.

## Figures and Tables

**Table 1:** Patient characteristics.

Characteristic	Discovery Cohort (*n* = 12)	Validation Cohort 1 (*n* = 60)	Validation Cohort 2 (*n* = 60)	*p*	Total Cohort (*n* = 132)
Age (years), median (range)	64 (49–78)	66 (41–81)	62 (49–79)	0.601	64 (41–81)
Sex, male (%)	7 (58.3)	34 (56.7)	36 (60.0)	0.934	77 (58.3)
Stage of disease (%) (Borderline) resectable Locally advanced Metastatic	2 (16.7) 4 (33.3) 6 (50.0)	29 (48.3) 18 (30.0) 13 (21.7)	31 (51.7) 23 (38.3) 6 (10.0)	0.016	62 (47.0) 45 (34.1) 25 (18.9)
Cycles of FOLFIRINOX received, median (range)	4 (2–12)	8 (2–12)	8 (1–12)	0.087	8 (1–12)
Baseline CA19-9 (kU/L), median (IQR)	410 (74.5–9341.0)	147.5 (51.8–910.3)	216.0 (51.0–845.0)	0.315	190.0 (51.0–1050.0)
RECIST response outcome after FOLFIRINOX ^a^ (%) Disease control Progressive disease	6 (50.0) 6 (50.0)	46 (76.7) 14 (23.3)	47 (78.3) 13 (21.7)	0.108	99 (75.0) 33 (25.0)

^a^ According to the RECIST 1.1 criteria. CA19-9 = carbohydrate antigen 19-9, IQR = interquartile range. *p*-values are calculated by Kruskal–Wallis tests (continuous data) or Chi-squared tests (categorical data).

**Table 2:** Differences in serum miRNA expression between pancreatic cancer patients with disease control and patients with progressive disease after FOLFIRINOX.

	Discovery Cohort (*n* = 6 DC, *n* = 6 PD)		Validation Cohort 1 (*n* = 46 DC, *n* = 14 PD)		Validation Cohort 2 (*n* = 47 DC, *n* = 13 PD)		Total Validation Cohort (*n* = 93 DC, *n* = 27 PD)	
miRNA	Log2 FD *	*p*	Log2 FD *	*p*	Log2 FD *	*p*	Log2 FD *	*p*
*Before start of FOLFIRINOX*								
hsa-let-7g-5p ^a^	−2.34	0.041	0.27	0.189				
hsa-miR-126-3p	−1.75	0.004	0.08	0.568				
hsa-miR-1290	1.78	0.039	0.28	0.451				
hsa-miR-17-3p	2.11	0.038	0.49	0.048	0.11	0.752	0.36	0.134
hsa-miR-194-5p ^a^	−2.44	0.015	0.57	0.112				
hsa-miR-199a-5p	−1.64	0.014	−0.34	0.254				
hsa-miR-200c-3p	3.52	0.032	0.48	0.185				
hsa-miR-30a-5p ^a^	−2.64	0.041	0.41	0.007				
hsa-miR-373-3p	8.37	<0.001	0.84	0.110	0.92	0.007	0.88	0.006
hsa-miR-629-5p	−3.56	0.048	0.58	0.015				
*After one cycle of FOLFIRINOX*								
hsa-let-7g-5p ^a^	−1.66	0.020	−0.09	0.570				
hsa-miR-18a-5p	−1.83	0.007	−0.32	0.027	0.56	0.016	0.13	0.361
hsa-miR-19a-3p	−1.72	0.049	−0.05	0.793				
hsa-miR-194-5p ^a^	−2.25	0.017	−0.50	0.026	−0.15	0.421	−0.29	0.044
hsa-miR-24-3p	−3.92	0.036	−0.78	0.024	0.53	0.073	−0.08	0.715
hsa-miR-27a-3p	−2.22	0.041	−0.95	0.008	0.45	0.208	−0.20	0.459
hsa-miR-30a-5p ^a^	−1.78	0.020	−0.16	0.235				
hsa-miR-30d-5p	−3.35	<0.001	−0.15	0.205				
hsa-miR-92b-3p	−1.94	0.049	0.16	0.563				

^a^ MicroRNAs were selected for validation in both samples before the start of FOLFIRINOX as well as samples after one cycle of FOLFIRINOX. DC = disease control, FD = fold difference, hsa = homo sapiens (human), miR/miRNA = microRNA, PD = progressive disease. * The disease control patient group was set as the reference group. *p*-values by t-tests.

**Table 3:** Univariable and multivariable binary logistic regression model for the prediction of early tumor progression during FOLFIRINOX.

	Univariable		Multivariable	
Variable	OR (95% CI)	*p*	OR (95% CI)	*p*
Stage of disease Resectable LAPC Metastatic	Ref 1.31 (0.58–2.95) 1.87 (0.65–5.35)	0.521 0.245		
CA19-9 at baseline (per 100 kU/L)	1.00 (1.00–1.01)	0.475		
miR-17-3p relative expression over reference miRNAs (per 1 × 10^−2^ increase) ^a^	1.39 (0.91–2.13)	0.125		
miR-373-3p relative expression over reference miRNAs (per 1 × 10^−2^ increase) ^a^	2.62 (0.90–7.63)	0.078	3.99 (1.10–14.49)	0.035
miR-194-5p relative expression over reference miRNAs (per 1 × 10^−2^ increase) ^b^	0.94 (0.87–1.00)	0.065	0.91 (0.83–0.99)	0.030

^a^ In samples before the start of FOLFIRINOX, ^b^ in samples after one cycle of FOLFIRINOX. CA19-9 = carbohydrate antigen 19-9, CI = confidence interval, OR = odds ratio, miR/miRNA = microRNA, Ref = reference.

**Table 4:** Univariable and multivariable Cox proportional hazards model for overall survival (OS) after FOLFIRINOX.

	Univariable		Multivariable	
Variable	HR (95% CI)	*p*	HR (95% CI)	*p*
Age (per year)	1.01 (0.99–1.04)	0.226		
Stage of disease Resectable LAPC Metastatic	Ref 1.00 (0.66–1.54) 2.16 (1.36–3.43)	0.985 0.001	Ref 1.40 (0.72–2.71) 2.51 (1.21–5.23)	0.316 0.014
CA19-9 at baseline (per 100 kU/L)	1.00 (1.00–1.01)	0.009	1.00 (1.00–1.01)	0.116
RECIST response outcome Disease control Progressive disease	Ref 3.85 (2.58–5.73)	<0.001	Ref 4.64 (2.48–8.68)	<0.001
miR-17-3p relative expression over reference miRNAs (per 1 × 10^−2^ increase) ^a^	1.30 (1.02–1.65)	0.032	1.18 (0.92–1.52)	0.192
miR-373-3p relative expression over reference miRNAs (per 1 × 10^−2^ increase) ^a^	1.15 (0.96–1.38)	0.141		
miR-194-5p relative expression over reference miRNAs (per 1 × 10^−2^ increase) ^b^	0.96 (0.90–1.02)	0.145		

^a^ In samples before the start of FOLFIRINOX, ^b^ in samples after one cycle of FOLFIRINOX. LAPC = locally advanced pancreatic cancer, CA19-9 = carbohydrate antigen 19-9, CI = confidence interval, HR = hazard ratio, miR/miRNA = microRNA, Ref = reference.

## Data Availability

The data presented in this study are available on request from the corresponding author.

## Supplementary Materials

The following are available online at https://www.mdpi.com/article/10.3390/ijms222010902/s1, Figure S1: In situ hybridization of liver, kidney, and pancreatic ductal adenocarcinoma (PDAC) liver metastasis tissue, Figure S2: In situ hybridization of positive control tissues with miR-373-3p, Table S1: Differences in serum miRNA expression between stages of disease, Table S2: Differences in serum miRNA expression between patients with disease control and patients with progressive disease after FOLFIRINOX for the three different stages of disease.
