# Supplementary material for: Serum miR-373-3p and miR-194-5p Are Associated with Early Tumor Progression during FOLFIRINOX Treatment in Pancreatic Cancer Patients: A Prospective Multicenter Study
Source: Int J Mol Sci. 2021 Oct 9;22(20):10902. doi: 10.3390/ijms222010902 (PMC8535910; doi:10.3390/ijms222010902)

Table S1. Differences in serum miRNA expression between stages of disease.

**miRNA**	**Resectable disease (*n*=60) Log2 FD**	**LAPC (*n*=41) Log2 FD**	**Metastatic disease (*n*=19) Log2 FD**	***P***
Before start of FOLFIRINOX				
hsa-miR-17-3p	Ref	-0.44	0.14	0.030
hsa-miR-373-3p	Ref	-0.29	-0.08	0.607
After one cycle of FOLFIRINOX				
hsa-miR-18a-5p	Ref	0.33	0.21	0.474
hsa-miR-194-5p	Ref	0.32	0.15	0.397
hsa-miR-24-3p	Ref	0.43	-0.03	0.198
hsa-miR-27a-3p	Ref	0.26	0.20	0.883

FD = fold difference, LAPC = locally advanced pancreatic cancer, hsa = homo sapiens (human), miR/miRNA = microRNA. *P*-values by one-way ANOVA.

Table S2. Differences in serum miRNA expression between patients with disease control and patients with progressive disease after FOLFIRINOX for the three different stages of disease.

**miRNA**	**Resectable disease (*n*=60) Log2 FD***	***P***	**LAPC (*n*=41) Log2 FD***	***P***	**Metastatic disease (*n*=19) Log2 FD***	***P***
Before start of FOLFIRINOX						
hsa-miR-17-3p	0.58	0.040	0.09	0.829	-0.60	0.459
hsa-miR-373-3p	-2.16	0.566	1.56	<0.001	0.31	0.721
After one cycle of FOLFIRINOX						
hsa-miR-18a-5p	-0.03	0.874	0.03	0.880	0.04	0.900
hsa-miR-194-5p	-0.42	0.173	-0.36	0.434	-0.60	0.254
hsa-miR-24-3p	-0.13	0.689	-0.18	0.651	-0.40	0.401
hsa-miR-27a-3p	-0.54	0.287	-0.37	0.534	-0.79	0.354

FD = fold difference, LAPC = locally advanced pancreatic cancer, hsa = homo sapiens (human), miR/miRNA = microRNA. *Fold differences are presented as the miRNA expression of progressive disease patients compared to disease control patients as reference group. *P*-values by t-tests.

Figure S1. In situ hybridization of liver, kidney, and pancreatic ductal adenocarcinoma (PDAC) liver metastasis tissue. Tissue sections are stained with U6 (positive control) or scramble miRNA probes (negative control), visualized with DAB. Scale bar = 100 µm.

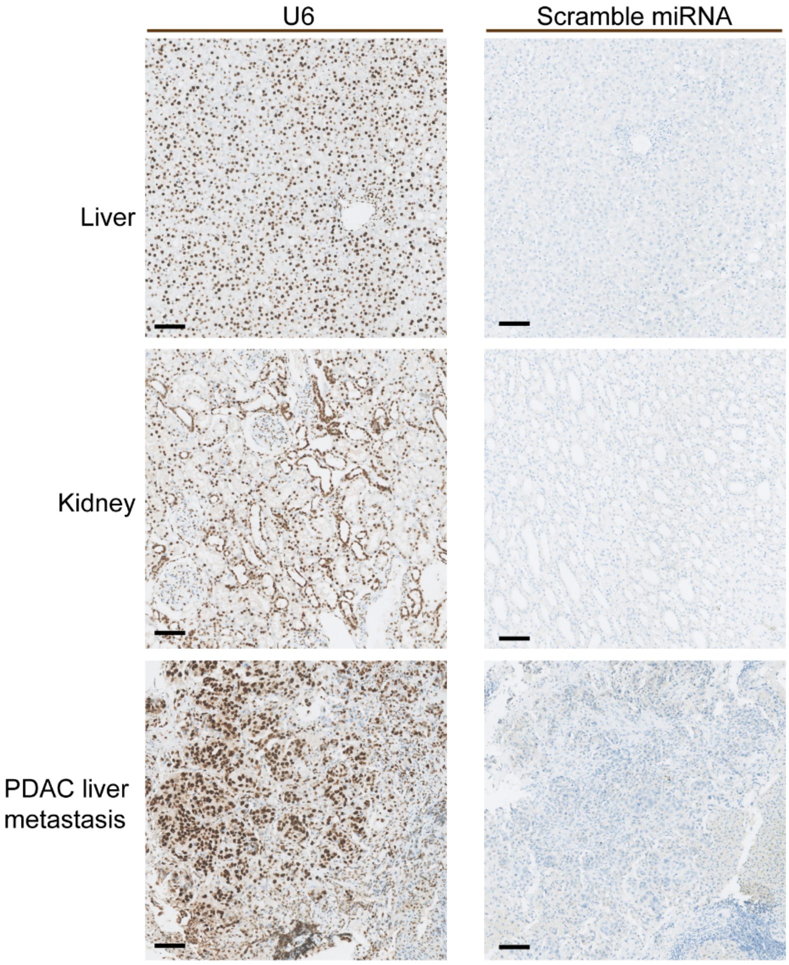

Figure S2. In situ hybridization of positive control tissues with miR-373-3p. Mir-373-3p expression is indicated in brown (DAB), cell nuclei are stained in blue with hematoxylin. MiR-373-3p is expressed by normal endothelium, hepatocytes, colon epithelium, renal tubular cells, neurons (brain), and in tonsillar B cell germinal centers. MiR-373-3p is also expressed in pancreatic acinar cells, but not in normal pancreatic ductal cells. Scale bar = 50 µm.

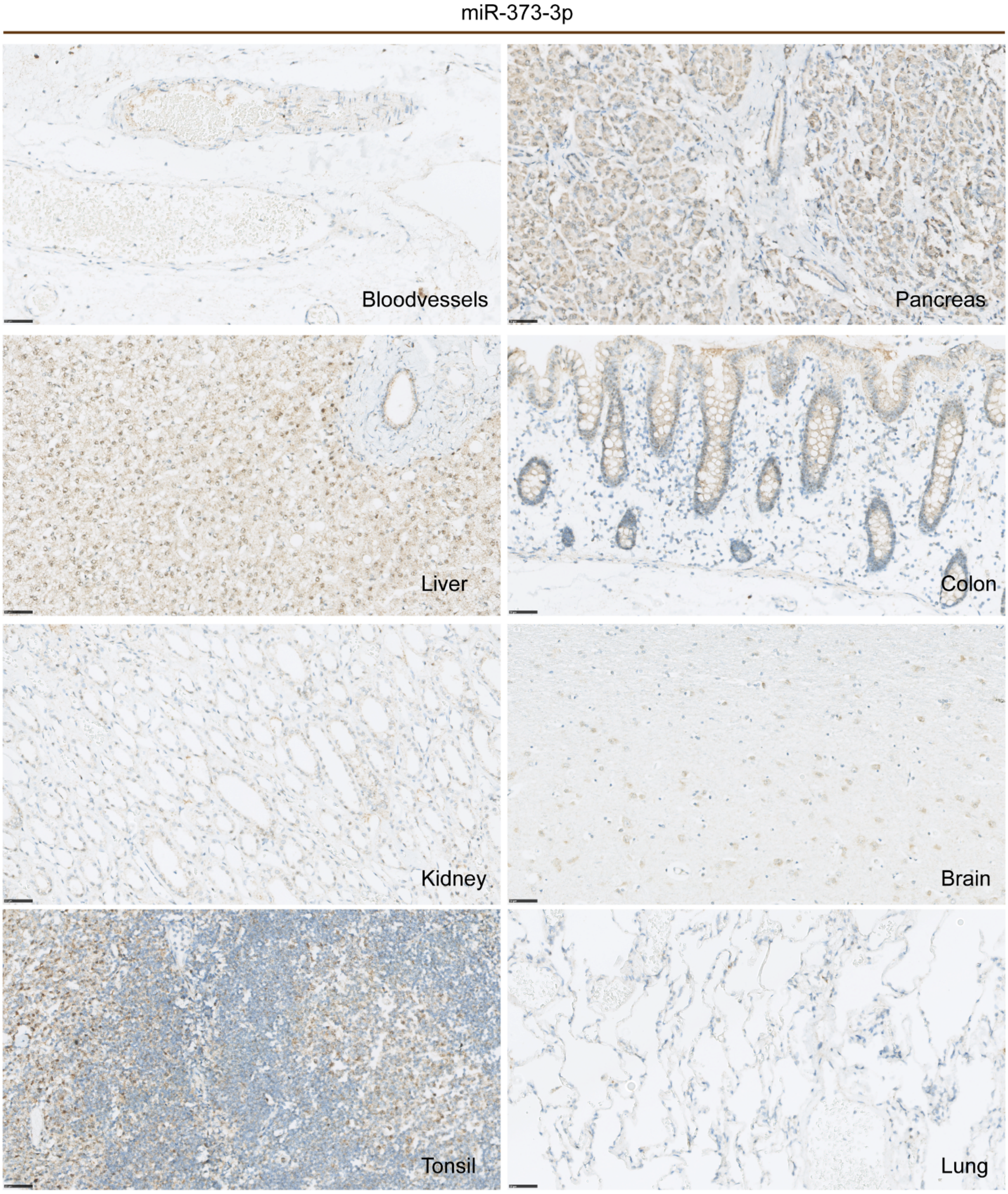

Supplement: Supplementary file 1 [file ijms-22-10902-s001.zip › ijms-1395011-supplementary.pdf]
